# Assessing spatial covariance among time series of abundance

**DOI:** 10.1002/ece3.2031

**Published:** 2016-03-12

**Authors:** Jeffrey C. Jorgensen, Eric J. Ward, Mark D. Scheuerell, Richard W. Zabel

**Affiliations:** ^1^ Conservation Biology DivisionNorthwest Fisheries Science CenterNational Marine Fisheries ServiceNational Oceanic and Atmospheric Administration2725 Montlake Blvd E.SeattleWashington98112; ^2^ Fish Ecology DivisionNorthwest Fisheries Science CenterNational Marine Fisheries ServiceNational Oceanic and Atmospheric Administration2725 Montlake Blvd E.SeattleWashington98112; ^3^ Present address: Ocean Associatesunder contract to Northwest Fisheries Science CenterNational Oceanic and Atmospheric Administration2725 Montlake Blvd E.SeattleWashington98112

**Keywords:** Chinook salmon, Columbia River, dynamic factor analysis, metapopulation, multivariate autoregressive state‐space models, population spatial structure, species diversity

## Abstract

For species of conservation concern, an essential part of the recovery planning process is identifying discrete population units and their location with respect to one another. A common feature among geographically proximate populations is that the number of organisms tends to covary through time as a consequence of similar responses to exogenous influences. In turn, high covariation among populations can threaten the persistence of the larger metapopulation. Historically, explorations of the covariance in population size of species with many (>10) time series have been computationally difficult. Here, we illustrate how dynamic factor analysis (DFA) can be used to characterize diversity among time series of population abundances and the degree to which all populations can be represented by a few common signals. Our application focuses on anadromous Chinook salmon (*Oncorhynchus tshawytscha*), a species listed under the US Endangered Species Act, that is impacted by a variety of natural and anthropogenic factors. Specifically, we fit DFA models to 24 time series of population abundance and used model selection to identify the minimum number of latent variables that explained the most temporal variation after accounting for the effects of environmental covariates. We found support for grouping the time series according to 5 common latent variables. The top model included two covariates: the Pacific Decadal Oscillation in spring and summer. The assignment of populations to the latent variables matched the currently established population structure at a broad spatial scale. At a finer scale, there was more population grouping complexity. Some relatively distant populations were grouped together, and some relatively close populations – considered to be more aligned with each other – were more associated with populations further away. These coarse‐ and fine‐grained examinations of spatial structure are important because they reveal different structural patterns not evident in other analyses.

“Given a species that is broken up into a number of such isolated groups or populations, it is obvious that the conservation of the species as a whole resolves into the conservation of every one of the component groups.” (Rich 1939)

## Introduction

Efforts to conserve at‐risk species can be hampered by a lack of understanding of the spatial structure of population units and the collective contributions of the populations to long‐term persistence and recovery (Hanski 1998; Rieman and Dunham 2000; Bowen and Karl 2007; Fullerton et al. 2011; Feldheim et al. 2014). The abundances of geographically proximate populations tend to fluctuate in concert with each other through time as a consequence of large‐scale exogenous influences (i.e., the “Moran effect”; Grenfell et al. 1998; Liebhold et al. 2004). However, diversity across populations and their habitats can result in a degree of asynchrony among populations. For example, checkerspot butterfly ecotypes that occupy diverse environments (coastal, low elevation, and interior high elevation) in California depend on different host plants that confer different levels of drought tolerance (Erlich et al. 1980). Individual populations of sockeye salmon in Alaska display a diversity of life history adaptations to variation in spawning and rearing habitats, which has sustained the metapopulation despite major changes in climatic conditions over the past century (Hilborn et al. 2003). In a cricket metapopulation, subpopulations that occupied small heterogeneous habitat patches weathered environmental stresses more successfully than those in large homogeneous habitat patches (Kindvall 1996).

Because of the importance of this spatial diversity in population response, there has been growing interest among researchers in portfolio theory (e.g., Schindler et al. 2010), a concept from finance applied to natural resource management (Figge 2004), where diversity in populations can lead to greater stability in a species. An important next step is to understand the mechanisms that maintain the population diversity (Ives and Carpenter 2007). In particular, can we identify the degree to which populations respond to common forcing functions and the degree to which they act independently? This type of information will be critical to manage populations across their spatial extent to maintain the persistence of at‐risk species.

Most efforts to understand population diversity have focused on population connectedness – examples include the analysis of DNA collected from individuals across the landscape (e.g., Guillot et al. 2005) or tagging data to quantify dispersal between populations (e.g., Block et al. 2005). More recently, researchers have focused on interpopulation diversity using multivariate time series analysis to quantify commonalities in observed indices of abundance (e.g., Ward et al. 2010; Ohlberger et al. In Press). This approach allows for a detailed examination of how populations covary through time and identification of common forcing functions that can be related to large‐scale environmental indicators. Here, we expand upon this approach and demonstrate an application to threatened Chinook salmon (*Oncorhynchus tshawytscha*, Walbaum 1792) to characterize population diversity. Pacific salmon are an interesting metapopulation example because of the high fidelity of spawners that return to their natal river tributary. This behavior promotes isolation and local adaptation of populations, and there is some level of spawner exchange via straying between populations, which maintains a connection among them (Policansky and Magnuson 1998; Rieman and Dunham 2000).

For anadromous Pacific salmonid species listed as threatened or endangered under the U.S. Endangered Species Act (ESA), understanding diversity – identifying population units, their spatial structure, and connectedness – has been an essential part of the recovery planning process (McElhany et al. 2000). They have been extensively studied, and several kinds of data have been used to describe population connectivity. The ESA listings cover broad domains, called evolutionarily significant units (ESUs; Waples 1991), each of which are composed of populations spread across relatively large geographic regions. The population structure is hierarchical, where at the broadest scale populations within an ESU are considered to be more similar to each other compared to populations outside of their ESU. Below the ESU level, several populations may be allied into distinct major population groups (MPGs; McElhany et al. 2000) within an ESU. Populations within such groupings may more frequently exchange individuals with each other than with populations in other MPGs or within the broader ESU. Below the population level, subpopulation structure may also exist (e.g., Quinn et al. 2006, 2012). Population persistence is, in part, a function of the degree of association and connectivity at these different levels (McElhany et al. 2000), and thus, assessing extinction risk requires a delineation of population structure and the status of constituent populations.

The salmon population delineation process has included the evaluation of molecular markers, ecological context (ecological community and landscape features where fishes spawn and rear), biogeography (river network structure, connectedness, and distance between tributaries), and phenotypic traits such as dates of migration or reproduction, and age and size at spawning (Waples et al. 2001; Ruckelshaus et al. 2002; ICTRT 2003). Comparisons of demographic data for assessing common and divergent characteristics have been limited, however, and therefore, the covariance of population size has played a lesser role in assessing population structure and diversity. Newly available demographic data and more accessible analytical techniques allow us the opportunity for assessing the spatial patterns of population diversity within and across ESUs. Specifically, we describe the spatial patterns of diversity among populations of spring‐ and summer‐run Chinook salmon in the interior Columbia River Basin, and we take advantage of this well‐studied system by comparing these spatial patterns to population structure determined through previous means (ICTRT 2003).

To do so, we rely on a time series technique called dynamic factor analysis (DFA; Zuur et al. 2003) rather than more common correlation analyses (e.g., Buonaccorsi et al. 2001; Liebhold et al. 2004). Interpretation of correlation analysis can be complicated by spatial and temporal autocorrelation that can lead to potentially false conclusions about the significance of correlations. DFA can explicitly account for the effects of exogenous drivers and covariance in the data when identifying common latent variables among the multiple time series.

## Materials and Methods

### Data

**Figure 1:**
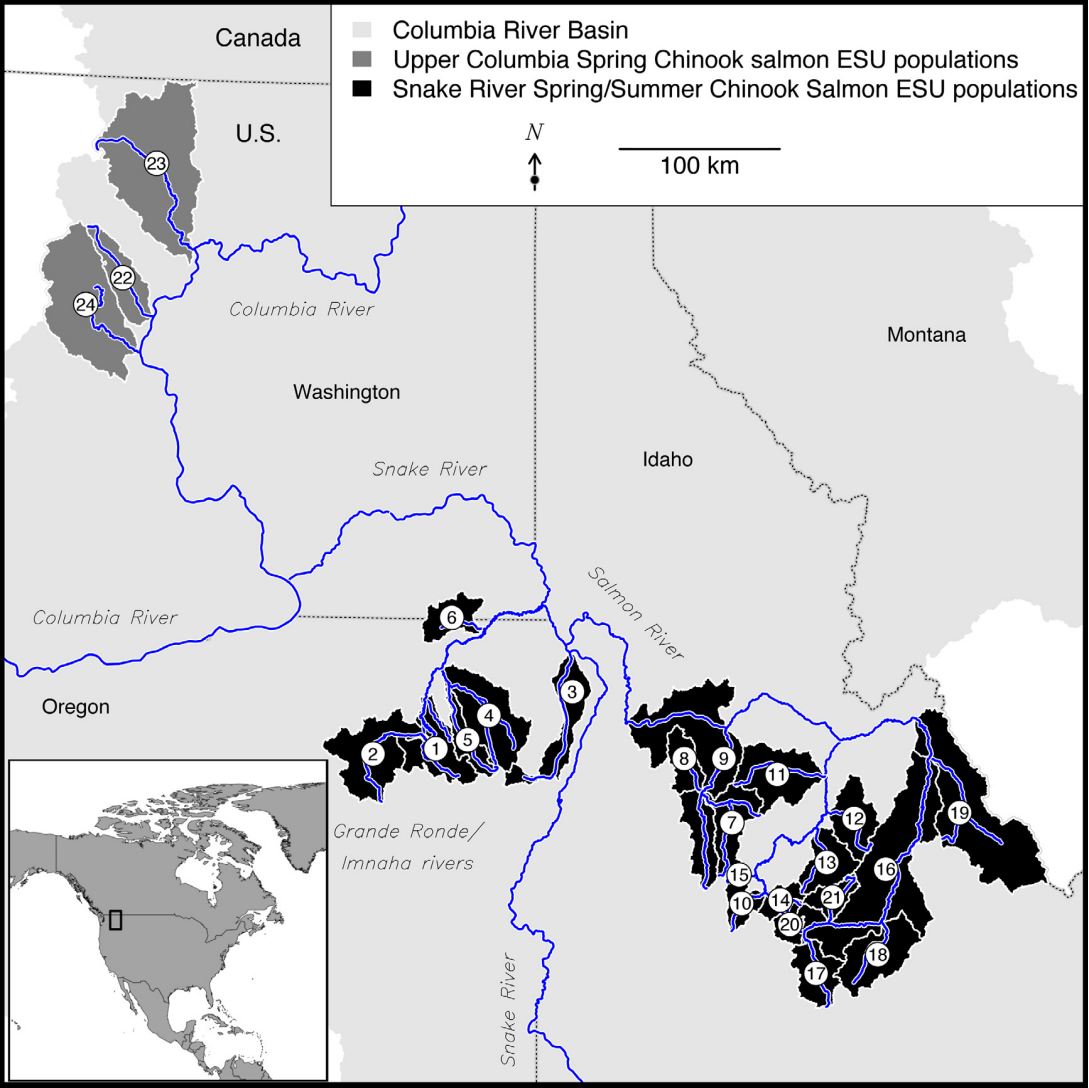
The Columbia River Basin, with populations included in this study (see Table S1). 1. Catherine Creek; 2. Upper Grande Ronde River; 3. Imnaha River; 4. Lostine River; 5. Minam River; 6. Wenaha River; 7. East Fork South Fork Salmon River; 8. Secesh River; 9. South Fork Salmon River; 10. Bear Valley Creek; 11. Big Creek; 12. Camas Creek; 13. Loon Creek; 14. Marsh Creek; 15. Sulphur Creek; 16. Lower Salmon River; 17. Upper Salmon River; 18. East Fork Salmon River; 19. Lemhi River; 20. Valley Creek; 21. Yankee Fork River; 22. Entiat River; 23. Methow River; 24. Wenatchee River.

The time series data we used in our analysis consisted of estimates of wild spawner abundance of spring‐ and summer‐run Chinook salmon populations from the interior Columbia River Basin (Fig. 1; Table S1). Chinook salmon are semelparous and spawn in freshwater streams. Offspring of Chinook salmon populations considered in this study have a juvenile life history characterized by rearing in freshwater until their second spring. Then, they migrate to the ocean and remain for 1–4 years before completing the return migration to their natal streams. Adult spawners consist mostly of 4‐year‐olds that spent 2 years in freshwater and 2 years at sea. Spawner estimates were derived from returning adult counts at weirs, redd (spawning nest) counts, and other sources of data (see Ford 2011; Salmon Population Summary (SPS Database 2015); Table S1). Data prior to 1957 were excluded because earlier years' estimates of abundance were available for only a few populations. Similarly, we discarded several populations from the analysis (Chamberlain Creek, and Pahsimeroi and Tucannon rivers) because only relatively recent years (since mid‐1980s) of spawner abundance estimates were available. There were 24 time series remaining, representing 2 ESUs. Abundance estimates of wild spawners (the yearly number of spawners includes wild and hatchery fish (if present), so this number was multiplied by the fraction of wild‐origin spawners) ranged from <10 individuals in some years to >4000 in other years for a few populations. Because the data were skewed (approximately log‐normally distributed), they were log‐transformed, and then, each time series was standardized to have zero mean and unit variance (this latter transformation was necessary because of assumptions about variances described below).

### Dynamic factor analysis

We used DFA to evaluate the data support for a range of possible underlying structures of population diversity. At the extremes, these structures range from one synchronous metapopulation to many independent subpopulations. This approach is analogous to traditional factor analysis and recently has become more accessible to ecologists (Zuur et al. 2003). The DFA approach is more flexible than assigning each observed time series to an underlying process (e.g., Ward et al. 2010), particularly when the number of time series is relatively large, such as the data set included in our analysis (*n *= 24).

The statistical model underlying DFA treats the observed data as linear combinations of one or more latent unobservable “trends” or variables, each of which is modeled as a random walk (Zuur et al. 2003). Latent variables that explain a large portion of the temporal variation in a particular time series receive large weight (positive or negative), while latent variables that explain minimal variation will receive a weight closer to zero. The treatment of unobservable latent variables makes DFA a conventional state‐space model (Clark and Bjørnstad 2004).

The salmon abundance data are well‐suited for use in DFA, as mentioned above: Adults return to freshwater from the ocean and are philopatric which segregates organisms into discrete units and selects for local adaptation and thus independence between populations; however, there is some exchange or straying of individuals between populations and as a consequence creates the potential for hierarchical connectedness between populations (McElhany et al. 2000).

We used the MARSS package for the R programming language, which fits linear multivariate autoregressive state‐space models with Gaussian errors (Holmes et al. 2013). Our parameterization of the DFA model included explanatory variables drawn from a set of environmental covariates. The model took this form:

(1a) $$y_{t}={\mathrm{Zx}}_{t}+{\mathrm{Dd}}_{t}+v_{t}$$

(1b) $$x_{t}=x_{t-1}+w_{t}$$

The vectors of the natural logarithms of *n* Chinook salmon spawner counts at time *t* (**y**_***t***_) were modeled as linear combinations of hidden latent variables, or trends, at time *t* (or states, **x**_***t***_) times factor loadings (**Z**), plus the effects of any exogenous covariate influence at time *t* (**Dd**_*t*_), plus some random observation errors **v**_***t***_, which were distributed as a multivariate normal with mean vector **0** and variance–covariance matrix **R**. The *m* latent variables at time *t* (**x**_*t*_) follow random walks governed by process errors **w**_*t*_, which were distributed as a multivariate normal with mean vector **0** and variance–covariance matrix **I**, which was set to an *m *× *m* identity matrix to ensure identifiability (Zuur et al. 2003; Holmes et al. 2012, 2013).

Starting with the hypothesis that all populations (*n *= 24 time series) exist as a single metapopulation (i.e., represent the observations of the same process), we fit models having from *m *=* *1 to many latent variables. We conducted estimation in a maximum‐likelihood framework (Holmes et al. 2013; R Core Team 2015). The data support for each model was quantified using the small‐sample Akaike information criterion (AICc; Burnham and Anderson 2002). After ensuring that parameter estimation converged, we examined model fits to data and examined potential problems with residuals.

We explored several structures for the **R** matrix, including allowing the populations to be independent (i.e., where only diagonal elements of **R** were estimated). Ultimately, comparisons of AICc scores suggested that there was little data support for these alternative forms relative to an **R** matrix that used two parameters: an equal element on the diagonal and an equal element in the off‐diagonals (i.e., the observation errors are identically distributed, but not independent; see Supporting Information for more details).

### Environmental covariates

After fitting models without covariates to identify a range of the more supported number of latent variables, we added environmental covariates to determine whether any of the underlying patterns and latent variables in the time series data could be better represented by including environmental drivers. Because anadromous Chinook salmon habitat spans a large range, from inland Columbia River tributary streams far from the coast to ocean habitats, we chose large‐scale covariates covering both freshwater and marine domains (Table S2). Our initial list of covariates was inspired by recent studies of potential environmental drivers of Pacific salmon population dynamics in multiple environments and was limited to indices with equivalent length to our longest salmon abundance time series. These included measures of snow water equivalent (SWE, or snowpack) on 1 April (a measure of accumulated winter mountain snowpack; Copeland and Meyer 2011), the Pacific Decadal Oscillation (PDO; Zabel et al. 2006; ICTRT and Zabel 2007; Rupp et al. 2012), El Niño (Hare et al. 1999), and Pacific Ocean coastal upwelling (Ryding and Skalski 1999; Botsford and Lawrence 2002; Logerwell et al. 2003; Scheuerell and Williams 2005). Accumulated winter snowpack measured in the spring can indicate low flow conditions in freshwater during summer that may stress fish, and PDO, El Niño, and coastal upwelling are generally more suggestive of conditions experienced by fish in the ocean.

We reduced the list of environmental covariates because several indices were highly correlated. To avoid multicollinearity, we used seasonal means (spring [April–June], summer [July–September], fall [October–December], winter [January–March]) for the PDO and coastal upwelling because adjacent monthly indices were relatively highly correlated (e.g., April with May, May with June). Because regional snowpack measures were highly correlated with each other, we created a comprehensive snowpack index which consisted of the mean of 9 snowpack measurement sites at three locations each from Washington, Oregon, and Idaho that are within or near each ESU (Table S2). Although there is some connection between El Niño, PDO, and freshwater conditions such as snowpack (e.g., McCabe and Dettinger 2002), we found weak correlations between the seasonal means, for example, of the PDO and snowpack (e.g., the strongest was with spring PDO, *r *=* *−0.43, with considerable variability).

We considered year lags as well as some additional derived ocean indices in our list of environmental covariates. Because the fish data consisted of spawner abundances of mixed‐age fish, we considered lags in ocean indices to correspond to ocean occupancy. Ocean entry for interior Columbia River spring/summer Chinook salmon populations typically occurs at a common age, but the number of years spent in the ocean varies. Thus, 4‐year‐old spawners in 1999 would have begun their life cycle in freshwater in 1995 and entered the ocean in 1997; therefore, the 1997 ocean index value was shifted to match the 1999 spawner abundance data. Although spawners are usually composed of a majority of 4‐year‐old fish, there are older and younger fish on the spawning grounds. To account for variability in spawner age, we considered additional lags of winter PDO covariates (Table S2). For the PDO, we included the spring and fall seasonal means for their ocean entry year and also included winter PDO values lagged to correspond to the first winter at sea. We also considered lags in snowpack. In addition, we included multiyear means of the May–July PDO, beginning from the year prior to spawning and extending backward in time for a total of 4 and 5 years as these were found to be a predictor of coastal coho salmon recruits (Rupp et al. 2012).

Before inclusion in the DFA model, we added a constant when needed to the environmental covariates to remove negative values and zeros, log‐transformed, and then standardized them (zero mean and unit variance) to remain consistent with the salmon abundance data transformations. We compared models fitted with transformed and untransformed covariates, and there was generally more data support for models with transformed covariates.

The DFA framework is flexible, and it allows each population to be affected by unique covariates, or by a common covariate whose impact differs by population. Because many of the environmental drivers in this analysis have an impact over a large spatial scale (e.g., PDO), we assumed that the covariates could impact each population, but the effect of each covariate on each population could vary. Therefore, each model contained additional *n* parameters for every covariate in the model. As an alternative parameterization, we evaluated a **D** matrix with shared effects of the covariates, which estimated one parameter that applied to all populations for each covariate added (i.e., each population is affected similarly).

### Mapping populations onto the common latent variables

Like principal components analysis, a challenge in applications of DFA is how loadings should be interpreted, in understanding both the estimated latent variables and how populations may be grouped based on their individual factor loadings with the estimated latent variables. Because the data were standardized prior to the analysis, the estimated latent variable values may be positive or negative. It is possible that a time series may be associated with a latent variable in a negative fashion (Zuur et al. 2003); thus, while for example the estimated latent variable may be moving upward, the data series may be trending downward. Zuur et al. (2003) demonstrated one approach to interpret potential grouping patterns of factor loadings, which consists of looking for grouping patterns according to the values of factor loadings for each latent variable. We include an additional method for latent variable associations. For each population *i* and latent variable *j*, we calculated the correlation between the vector of observed data after controlling for the environmental covariate effects (i.e., the *i*th row of **y** – **Dd**) and each of the latent variables (i.e., the *i*th row and *j*th column of **Z** times the *i*th row of **x**). The highest positive correlations among the latent variables determined the latent variable associations for each population.

## Results

**Figure 2:**
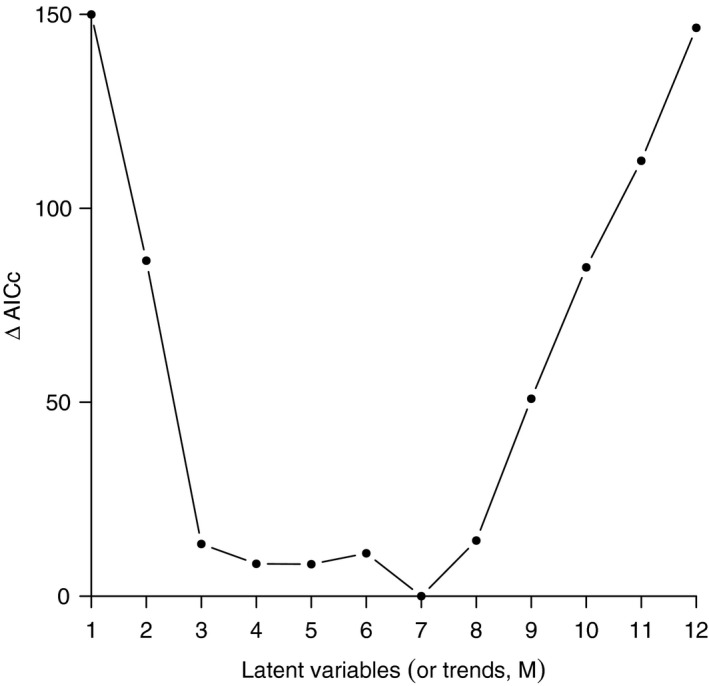
Delta (Δ) AICc values as a function of the number of latent variables (or trends) from models fitted without environmental covariates. The Δ AICc values are relative to the AICc score of the model with 7 latent variables or trends.

**Figure 3:**
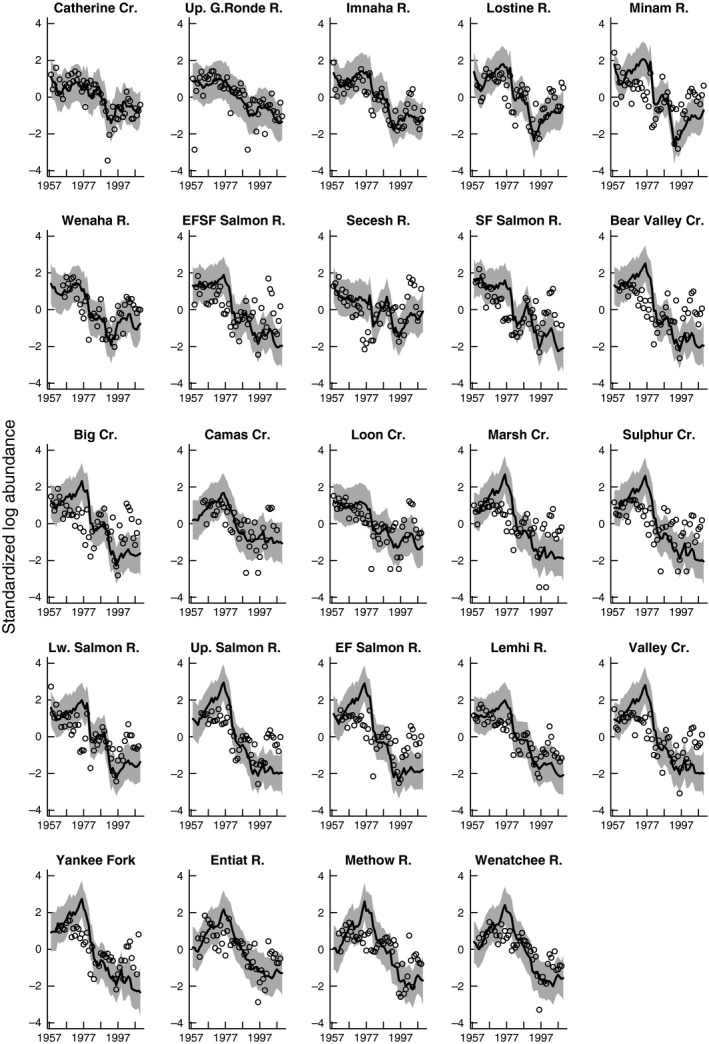
Model fits (lines) versus observed spawner abundance data (open circles), and shading indicates 95% confidence intervals of the model fits.

Initial analyses to identify the range of latent variables within which we should target further analyses suggested more data support for models with 4 to 7 common latent variables among the 24 populations of Chinook salmon time series (Fig. 2). We focused on subsequent model fitting by including each of the environmental covariates individually to DFA models with 4 to 7 latent variables. The ranked order of these models, according to their AICc scores, determined the order in which covariates were added (Table S3). In the next step, covariates were added to models with 4 to 7 latent variables according to their rank in a forward stepwise procedure because computational constraints prevented an exhaustive model search. The model with the most support from the data was a 5‐latent variable model that included spring and summer PDO (the means of April–June and July–September values) lagged 3 years (Table 1; Fig. 3). The 3‐year lag corresponds to the spring and summer juvenile freshwater rearing period for 4‐year‐old spawners and for 5‐year‐old spawners to the period of juvenile out‐migration to the ocean and the early marine period during the first summer at sea.

**Table 1:** The top 15 DFA models with the most support from the data. The “M” values are the number of latent variables, and “K” represents the number of parameters estimated. Environmental covariates consisted of seasonal Pacific Decadal Oscillation (“pdo”), an index of mountain snowpack on 1 April (“snotelall”), seasonal coastal ocean upwelling (“up”), and El Niño 3.4 index, with lags between 0 and 3 years (e.g., L3). Environmental covariates tagged with an asterisk, “*,” indicate model parameterizations where the terms in the **D** matrix were set to estimate one coefficient across all populations when that covariate was added to the model. See Table S2 for sources, derivation, and further explanation of the environmental covariates, and see Table S3 for covariate inclusion order. Akaike weights were calculated on AICc

M	K	Environmental covariates	Δ AICc	Akaike weight
5	137	pdoJASL3, pdoAMJL3*	0	0.427
5	136	pdoJASL3	1.4	0.207
4	117	pdoJASL3, pdoAMJL3*	2.6	0.116
4	116	pdoJASL3	3.4	0.077
7	150	pdoJASL2*	5.7	0.025
6	156	pdoJASL3, pdoAMJL3*	5.9	0.022
7	150	pdoAMJL2*	6.5	0.017
7	150	upAMJL2*	7.0	0.013
7	150	elninoL2*	7.2	0.012
7	150	pdoAMJL3*	7.2	0.011
7	150	pdoJFML4*	7.5	0.01
7	150	pdoONDL2*	8.1	0.007
7	149	None	8.7	0.005
7	150	pdoJASL3*	9.2	0.004
7	150	snotelallL2*	9.6	0.004

**Figure 4:**
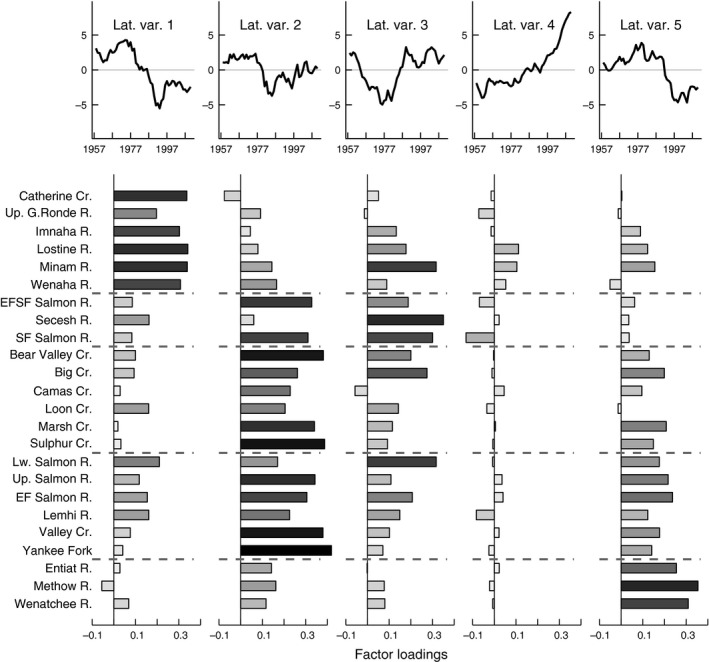
Latent variables (top row) and factor loadings per latent variable for the best‐fitting model, which included five latent variables and spring and summer PDO (Table 1). Bars for the factor loadings are filled in gray scale according to their absolute value (near 0 = white, ±1 = black), where their values indicate strength of latent variable association. The horizontal dashed lines mark major population groups (Table S1).

The combination of the loadings and latent variables plus the effect of any exogenous drivers provides the overall model fits for each population (Figs. 3 and 4). These model fits allow us to classify the entire 50‐year time series into three general periods of population growth (Fig. 4). The first 20 years were marked by similar decreases in abundance among all populations. The next 20 years were much more variable and included a combination of further declines and relative stability. The last 10 years generally saw stable or increasing trends in abundance.

There was concordance of population groupings at the broad ESU‐level spatial scale with the existing delineations, but at the MPG level (which is a finer spatial scale), we found some differences. At the broader ESU level, populations in the Upper Columbia and the Snake River ESUs grouped separately from each other which is consistent with the current population structure at this spatial scale (ICTRT 2003). Upper Columbia ESU populations (Methow, Entiat, and Wenatchee rivers) had the most association only with latent variable 5 (Fig. 4), and Snake River ESU populations were associated with latent variables 1–4. Within the Snake River ESU, at the finer MPG level, there were several populations that were more associated with an MPG different from their putative MPG groupings. Specifically, factor loadings (Fig. 4) per latent variable exhibited a few clear patterns: Most Grande Ronde/Imnaha MPG populations were closely aligned with latent variable 1; however, a few populations had some association with latent variable 3; the three South Fork Salmon River MPG populations were more associated with latent variables 2 and 3; and the remainder of the Salmon River MPG populations (Upper, Middle Fork, and South Fork Salmon River MPGs) were generally associated with latent variables 2 and 3.

**Figure 5:**
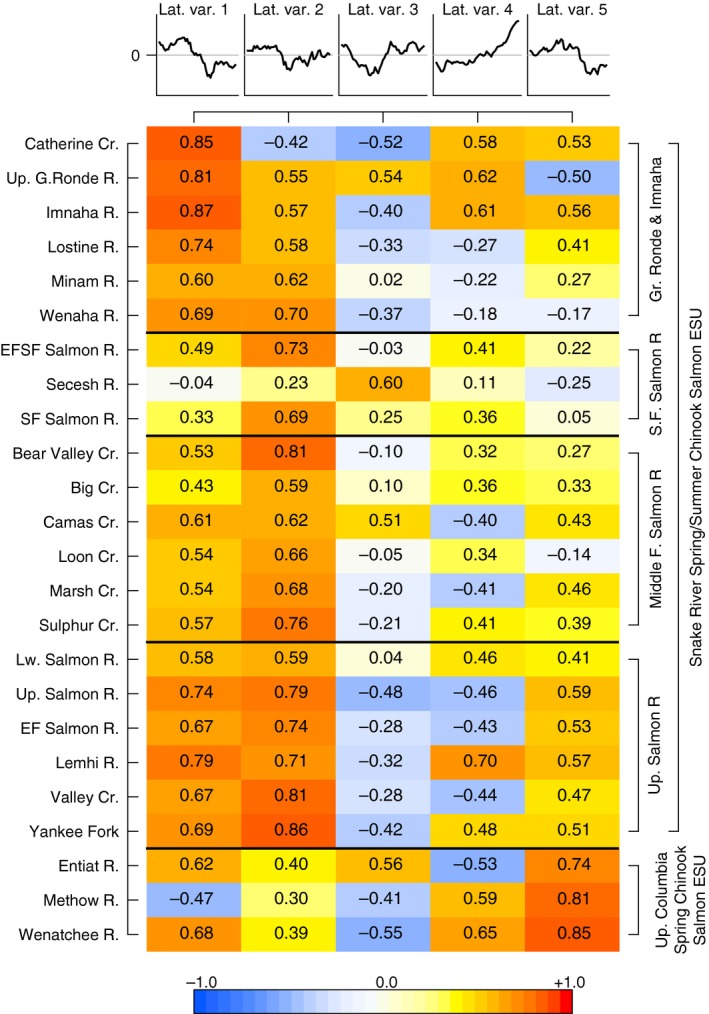
Correlations between the data, corrected for covariate effects, and latent variables.

**Figure 6:**
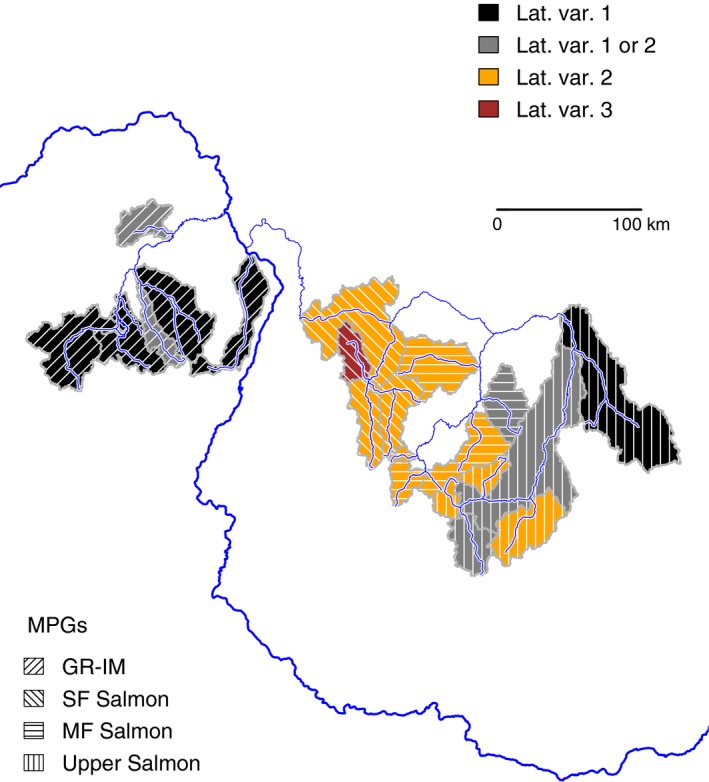
Spatial representation of Snake River Spring Summer Chinook salmon ESU populations grouped according to the results of the latent variable associations from the correlations between the data, minus the covariate effects, and the top model's fits (from Fig. 5). The hatched lines indicate the population groupings into MPG assignments according to the currently identified boundaries (ICTRT 2003), and colors represent population groupings according to correlations with the latent variables (from Fig. 5).

The population grouping method using the correlations between time series data, minus the covariate effects, and the latent variables had the same pattern as just looking at factor loadings at the broad ESU scale grouping pattern between the Upper Columbia and Snake River populations, and similar but different latent variable association patterns at the within‐ESU finer spatial scale (Fig. 5). At the finer scale, some populations within an MPG were associated with one latent variable, and others appeared to be nearly equivalently associated with two latent variables, and in one case, one population appeared to be the only one associated with one latent variable. Four of the six Grande Ronde and Imnaha MPG populations were more clearly associated with latent variable 1, while the other two were linked to latent variable 2. A majority of populations in the Salmon River MPGs (Upper Salmon River MPG, Middle Fork Salmon River MPG, South Fork Salmon River MPG) were associated with latent variable 2, but a few populations were closely aligned with either latent variable 1 or 2. And one population in the Upper Salmon River MPG, the Lemhi River, was more closely associated with latent variable 1 – characteristic of the Grande Ronde/Imnaha MPG populations and which is >500 km away (Figs. 5 and 6; Table S5). The Upper Salmon River MPG populations appeared to be closely related to either latent variable 1 or 2. Among the Grande Ronde/Imnaha MPG populations, both the Wenaha and Minam rivers' populations were closely associated with both latent variables 1 and 2. These populations are at least 250 km distant from the nearest populations in the Salmon River Basin (Table S4) which also had many populations associated with both latent variables 1 and 2. In contrast to the grouping by factor loadings (Fig. 4), where several populations had strong associations with latent variable 3, in the correlations method the Secesh River (South Fork Salmon River MPG) population grouped separately from all other populations considered in this study and was the only population with the strongest association to latent variable 3 (Fig. 5).

## Discussion

Our analysis of Chinook salmon populations from the Columbia River revealed population dynamics patterns across the landscape that were both expected and unexpected. At the broad geographic scale, populations exhibited distinct temporal patterns of abundance. For example, Upper Columbia Evolutionarily Significant Unit (ESU, the unit of conservation under the Endangered Species Act) populations were never grouped with Snake River ESU populations (between 500 and 1000 km apart). However, when we examined population dynamics at the scale of the major population group (MPG, groupings of related populations within ESUs), the patterns were more complex, as described below. This type of information is critical for understanding population dynamics and can provide important information for managing these at‐risk populations.

At the broader geographic scale, drivers of population dynamics were distinct and strong enough to produce distinguishable population responses when comparing across ESUs. Factors that might explain these differences include differences in ocean survival (e.g., Sharma et al. 2013), separate migratory corridors, and differences in climate and geology.

We found that the grouping patterns were more complex at a finer spatial scale where distances between populations are shorter. For populations within the Snake River ESU, several pairs of populations were found to group together despite the relatively large distances separating them: for example, Lemhi River with the Grande Ronde populations. This suggests that some other factors may have been responsible for linking these populations, such as a common response to an exogenous driver(s) not captured in our analyses. Both sets of populations traverse the same set of main stem dams going to and returning from the ocean. Generally, the Lemhi basin is drier, has cooler surface air temperatures (particularly in winter), and is higher in elevation than the Grande Ronde and Imnaha basins (basin attributes are summarized in ICTRT 2003). All of the Grande Ronde populations have been supplemented to some degree by hatchery fish, and during the time span of our analysis, some hatchery fish have been permitted to spawn naturally, whereas the Lemhi population has not been supplemented (Ford 2011). Offspring from hatchery‐origin fish spawning in the wild have demographic impacts on subsequent years of the natural population although the extent of the impact appears to be mixed (Christie et al. 2014; Scheuerell et al. 2015). Further research is needed to reveal the underlying causes for similarities in latent variable associations in relatively distant populations within this ESU, whether from hatchery contributions or from other factors.

While a few relatively distant populations grouped together, there were cases where relatively geographically close populations grouped separately from one another. For example, the Secesh River population was more associated with a different latent variable than the other two populations in the South Fork Salmon River MPG, a latent variable to which none of the other study populations was closely linked. This could be indicative of demographic isolation or independence. There could be several mechanisms responsible for this lone latent variable association, one of which could be alternative juvenile life history rearing strategies. Each year some proportion of juveniles leave their natal streams early and rear in downstream habitats in response to habitat and environmental conditions and perhaps other factors, and the Secesh River population is among those with a high proportion of downstream‐rearing juveniles (Copeland et al. 2014). These types of divergent latent variable association patterns may represent important components to MPG, ESU, and species diversity which may have implications for setting conservation priorities: specifically, prioritizing which populations to target for recovery efforts (A. Fullerton, S. Anzalone, D. Van Doornik, T. Copeland, P. Moran, and R. Zabel, In Review.).

In some instances, populations appeared somewhat equally grouped with multiple latent variables, depending on the grouping method, a few of which were more associated with populations outside of their MPG. The grouping patterns were less complex in the correlations method, where a majority of the Snake River ESU populations were associated with either latent variable 1 or 2, or in a few cases, both. Five of the Snake River ESU populations (Minam River, Wenaha River, Camas Creek river, and Lower and Upper Salmon rivers) had nearly equal associations with both latent variables. Snake River ESU population groupings by factor loadings were more complex. For example, although all Grande Ronde/Imnaha MPG populations had strong associations with the first latent variable, a few of the populations in this MPG also had strong associations with another latent variable. External influences such as hatchery strays could potentially confound unique latent variable association, and although hatchery strays have been documented in this MPG, they have likely contributed very little to the abundance dynamics of these populations over time (Van Doornik et al. 2013). Because of these pattern differences between grouping methods, it is probably prudent to implement several grouping methods and to consider other population data when interpreting results.

Each grouping method suggested some evidence for alternative population associations compared to the delineations currently recognized, and population assignment to MPGs has important conservation implications. For example, in the interior Columbia Basin assessment of the viability of Chinook salmon is carried out hierarchically, starting first with ratings of viability of each population‐level unit. A viability metric for an MPG is determined from its constituent populations' viability ratings (McElhany et al. 2000; ICTRT 2007). Thus, knowing the MPG to which a population should be assigned has ramifications for an MPG's measure of viability and consequently in the hierarchy to the ESU level.

For salmon, expression of multiple life history characteristics, dispersal (gene flow), phenotypic plasticity, and capacity for rapid evolution are thought to be important contributors to species stability and resiliency (Waples et al. 2009). Aspects of these attributes are often included as important characteristics for persistence of many species (e.g., Maron et al. 2004; Yeaman and Jarvis 2006; Kerr and Secor 2012). Results from a DFA analysis performed on abundance data paired with a similar analysis on genetic data such as allele frequencies over time from the same set of populations might prove to be useful metrics to quantify species diversity and to infer metapopulation structure.

Inclusion of environmental covariates in the DFA modeling was strongly supported by the data. All of the top 12 models (0 to 8.1 Δ AICc units; Table 1) included at least one covariate. The highest‐ranked model included spring and summer PDO (means of April–June and July–September) lagged 3 years. The PDO is a long‐frequency large basin‐scale environmental driver. It has been associated with the marine environment affecting salmon (Mantua et al. 1997) and to some extent with freshwater conditions such as snowpack (Clark et al. 2001; McCabe and Dettinger 2002) and, therefore, to late summer runoff which impacts summer flows for snowmelt‐dominated river systems. We found a correlation between our index measure of snowpack and the PDO. Associations with both the freshwater and marine environments may explain why the PDO appeared in the best‐supported model. The typical life history of these populations consists of a majority of 4‐ and 5‐year‐old spawners that spend nearly 2 years in freshwater before migrating seaward. Therefore, summer PDO lagged 3 years would correspond to 4‐year‐old spawners' first rearing summer in freshwater, a season when streamflows are typically at their lowest and when streamflow is sensitive to the amount of warm‐season melting of mountain snowpack from the previous winter season. For 5‐year‐old spawners, a 3‐year PDO lag would coincide with conditions during their first summer at sea. Both of these life stages (freshwater summer rearing and the early ocean period) are often cited as important population drivers (e.g., Crozier et al. 2008). Some evidence suggests that there are several other modes of climate variability more related to Pacific Northwest mountain snowpack levels than the PDO and that are not necessarily associated with each other or with the PDO (Stoelinga et al. 2010). Further exploration of additional environmental drivers may lead to additional insides into abundance covariation and population associations.

A number of different approaches have been proposed to help ecologists and wildlife managers identify and quantify the population association and connectedness. Many of these approaches, such as genetic analyses (Pritchard et al. 2000) or tagging data (Robichaud and Rose 2004), may be cost‐prohibitive to collect for organisms that exist on a large spatial or temporal scale, particularly if spatial or temporal variation is important in explaining population dynamics. In our analysis, we demonstrate how DFA may be a useful tool in characterizing covariance in population abundance, species diversity, and can provide some information about metapopulation structure from time series of population counts. DFA has been used to identify how different species correlate with environmental drivers (Zuur et al. 2003; Stachura et al. 2014) and to confer some information about species diversity and metapopulation structure (Ohlberger et al. In Press). There are several advantages of using the DFA framework over other time series methods (e.g., Ward et al. 2010). First, the number of time series may be prohibitively large to do exhaustive model selection, and second, the relationships between some populations within a metapopulation structure may not be black and white, but instead may exist in a gray area. Although we apply DFA to population counts, the general framework is applicable to virtually any type of temporal data for any species – similar analyses could be performed on phenotypic measurements from individuals (e.g., body size), reproductive success, or abiotic environmental variables (e.g., water temperature, contaminant data).

## Conflict of Interest

None declared.

## Data Accessibility

The abundance data used in this analysis came from the Salmon Population Summary database and are publicly available: https://www.webapps.nwfsc.noaa.gov/apex/f?p=261:home:0

## Supporting information


**Table S1.** Interior Columbia River Chinook salmon populations included in this study.
**Table S2.** Sources for the environmental covariates.
**Table S3.** Environmental covariates included in DFA model fitting.
**Table S4.** Stream network distances between populations.
**Table S5.** Stream network distances between Major Population Groups.

**Data S1.** R scripts illustrating DFA model fitting.
