# Supplementary material for: Assessing spatial covariance among time series of abundance
Source: Ecol Evol. 2016 Mar 12;6(8):2472–85. doi: 10.1002/ece3.2031 (PMC4789304; doi:10.1002/ece3.2031)
Supplement: Supplementary file 1 — Table S1. Interior Columbia River Chinook salmon populations included in this study. Table S2. Sources for the environmental covariates. Table S3. Environmental covariates included in DFA model fitting. Table S4. Stream network distances between populations. Table S5. Stream network distances between Major Population Groups. [file ECE3-6-2472-s001.docx]

# Supporting Information

**Table S1**: Interior Columbia River Chinook salmon populations (name codes in parentheses) included in this study.

| **Evolutionarily Significant Unit** | **Major Population Group** | **Population** | **Years** |
| --- | --- | --- | --- |
| Snake River Spring/Summer | Grande Ronde-Imnaha | Catherine Creek (GRCAT) | 1957-2009 |
| Chinook Salmon ESU |  | Grande Ronde River Upper Mainstem (GRUMA) | 1957-2009 |
|  |  | Imnaha River Mainstem (IRMAI) | 1957-2009 |
|  |  | Lostine River (GRLOS) | 1959-2009 |
|  |  | Minam River (GRMIN) | 1957-2009 |
|  |  | Wenaha River (GRWEN) | 1964-2009 |
|  |  |  |  |
|  | South Fork Salmon River | East Fork South Fork Salmon River (SFEFS) | 1958-2008 |
|  |  | Secesh River (SFSEC) | 1957-2008 |
|  |  | South Fork Salmon River Mainstem (SFMAI) | 1958-2008 |
|  |  |  |  |
|  | Middle Fork Salmon River | Bear Valley Creek (MFBEA) | 1960-2008 |
|  |  | Big Creek (MFBIG) | 1957-2008 |
|  |  | Camas Creek (MFCAM) | 1963-2008 |
|  |  | Loon Creek (MFLOO) | 1957-2008 |
|  |  | Marsh Creek (MFMAR) | 1957-2008 |
|  |  | Sulphur Creek (MFSUL) | 1957-2008 |
|  |  |  |  |
|  | Upper Salmon River | Lower Salmon River (SRLMA, below Redfish Lake) | 1957-2008 |
|  |  | Upper Salmon River (SRUMA, above Redfish Lake) | 1962-2008 |
|  |  | East Fork Salmon River (SREFS) | 1960-2008 |
|  |  | Lemhi River (SRLEM) | 1957-2008 |
|  |  | Valley Creek (SRVAL) | 1957-2008 |
|  |  | Yankee Fork (SRYFS) | 1961-2008 |
|  |  |  |  |
| Upper Columbia Spring |  | Entiat River (UCENT) | 1960-2008 |
| Chinook salmon ESU |  | Methow River (UCMET) | 1960-2008 |
|  |  | Wenatchee River (UCWEN) | 1960-2008 |

**Table S2**: Environmental covariates evaluated for inclusion in the DFA models with lags as related to four and five year old spawning fish.

| **Covariate** | **Description** |
| --- | --- |
| snotelall; L0, L1, L2 | 1 April SWE index^a^, lagged *t* – 0 up to *t* – 2 |
| pdoJFM; L3 & L4 | Pacific Decadal Oscillation^b^ (PDO) in winter (Jan-Mar mean), first (*t* – 3) winter at sea, and for five year old fish second (*t* – 4) winter at sea |
|  |  |
| pdoAMJ; L2, L3, L4 | PDO in spring (Apr-Jun mean), starting with first year in ocean (*t* – 2), included additional lags (*t* – 3, *t* – 4) |
|  |  |
| pdoJAS; L2, L3 | PDO in summer (Jul-Sept mean), starting with the first year in ocean (*t* – 2), for five year old fish included additional lag, *t* – 3. |
|  |  |
| pdoSept; L1, L2, L3 | PDO for September only, lagged from *t* – 1 up to *t* – 3 |
| pdoOND; L2, L3, L4 | PDO in fall (Oct-Dec mean), starting with first year in ocean (*t* – 2), included two additional lags (*t* – 3, *t* – 4) |
|  |  |
| pdo4yrMJJ | Mean of four year May-June PDO beginning in year prior to spawning year backward |
|  |  |
| pdo5yrMJJ | Mean of five year May-June PDO, beginning in spawning year backward |
|  |  |
| elnino; L0, L1, L2, L3 | El Niño 3.4 index^c^, lagged *t* – 0 up to *t* – 3 |
| upAMJ; L2 | Pacific Upwelling Index^d^ (PUI; 45°N 125°W) Apr-Jun mean, spring of ocean entry, lagged *t* – 2 |
|  |  |
| upOND; L2 | PUI Oct-Dec mean, fall after spring ocean entry (lagged *t* – 2) |
| upwellaprL2 | PUI for April only, lagged *t* – 2, spring of ocean entry |

Sources:

^a^Snow water equivalent (SWE) on 1 April, mean of nine snow telemetry (SNOTEL) and snow course sites: Washington, 20A23S Lyman Lake, 20A09S Rainy Pass, 19A02S Salmon Meadows; Idaho, 14E01S Mill Creek Summit, 13D16S Moose Creek, 14F02S Stickney Mill; Oregon, 18D06S Lucky Strike, 18E08S Gold Center, 18E03S Eilertson Meadows. Natural Resources Conservation Service; <http://www.wcc.nrcs.usda.gov/snow/>

^b^University of Washington-Joint Institute for the Study of the Atmosphere and Ocean; http://jisao.washington.edu/pdo/PDO.latest

^c^El Niño data from the NOAA National Weather Service Climate Prediction Center, <http://www.cpc.ncep.noaa.gov/data/>

^d^NOAA Pacific Fisheries Environmental Laboratory; <http://www.pfeg.noaa.gov/products/PFEL/modeled/indices/upwelling/NA/data_download.html>

**Table S3**: Covariates included in DFA model fitting, in order of inclusion in a forward step-wise procedure. Descriptions and sources are as in Table S2. An “*” next to a covariate indicates that the covariate’s effect was shared for all populations.

| **Order of inclusion** | **Covariate** | **Description** |
| --- | --- | --- |
| 1 | pdoJAS; L3 | Summer PDO lagged 3 yr |
| 2 | pdoAMJ*; L3 | Spring PDO lagged 3 yr |
| 3 | pdoOND; L3 | Fall PDO lagged 3 yr |
| 4 | pdoJAS*; L2 | Summer PDO lagged 2 yr |
| 5 | elnino* | El Niño index, no lag |
| 6 | upAMJ*; L2 | Spring coastal upwelling lagged 2 yr |
| 7 | elnino*; L2 | El Niño index lagged 2 yr |
| 8 | upOND*; L2 | Fall upwelling lagged 2 yr |
| 9 | pdoJAS*; L3 | Summer PDO lagged 3 yr |
| 10 | pdoOND*; L2 | Fall PDO lagged 2 yr |
| 11 | snotelall*; L2 | April 1st SWE index lagged 2 yr |
| 12 | pdoAMJ*; L2 | Spring PDO lagged 2 yr |
|  |  |  |

**Table S4**: Geographic stream network distances (km; adapted from ICTRT 2003) of the populations considered in this study.

|  |  | **Grande Ronde/Imnaha MPG** | | | | | | **SF Salmon MPG** | | | **MF Salmon MPG** | | | | | | **Upper Salmon MPG** | | | | | |
| --- | --- | --- | --- | --- | --- | --- | --- | --- | --- | --- | --- | --- | --- | --- | --- | --- | --- | --- | --- | --- | --- | --- |
|  |  | **GRWEN** | **GRLOS** | **GRMIN** | **GRCAT** | **GRUMA** | **IRMAI** | **SFMAI** | **SFSEC** | **SFEFS** | **MFBIG** | **MFCAM** | **MFLOO** | **MFSUL** | **MFBEA** | **MFMAR** | **SRLEM** | **SRLMA** | **SREFS** | **SRYFS** | **SRVAL** | **SRUMA** |
| **Grande Ronde/Imnaha MPG** | **GRWEN** | --- |  |  |  |  |  |  |  |  |  |  |  |  |  |  |  |  |  |  |  |  |
|  | **GRLOS** | 110 | --- |  |  |  |  |  |  |  |  |  |  |  |  |  |  |  |  |  |  |  |
|  | **GRMIN** | 114 | 55 | --- |  |  |  |  |  |  |  |  |  |  |  |  |  |  |  |  |  |  |
|  | **GRCAT** | 106 | 79 | 83 | --- |  |  |  |  |  |  |  |  |  |  |  |  |  |  |  |  |  |
|  | **GRUMA** | 224 | 197 | 201 | 129 | --- |  |  |  |  |  |  |  |  |  |  |  |  |  |  |  |  |
|  | **IRMAI** | 178 | 266 | 270 | 262 | 380 | --- |  |  |  |  |  |  |  |  |  |  |  |  |  |  |  |
| **SF Salmon MPG** | **SFMAI** | 258 | 347 | 351 | 342 | 461 | 206 | --- |  |  |  |  |  |  |  |  |  |  |  |  |  |  |
|  | **SFSEC** | 385 | 473 | 477 | 469 | 587 | 333 | 126 | --- |  |  |  |  |  |  |  |  |  |  |  |  |  |
|  | **SFEFS** | 386 | 474 | 478 | 470 | 588 | 334 | 128 | 1 | --- |  |  |  |  |  |  |  |  |  |  |  |  |
| **MF Salmon MPG** | **MFBIG** | 412 | 500 | 504 | 496 | 614 | 360 | 153 | 143 | 144 | --- |  |  |  |  |  |  |  |  |  |  |  |
|  | **MFCAM** | 487 | 575 | 579 | 571 | 689 | 435 | 229 | 218 | 219 | 75 | --- |  |  |  |  |  |  |  |  |  |  |
|  | **MFLOO** | 503 | 592 | 596 | 588 | 706 | 452 | 245 | 234 | 236 | 92 | 17 | --- |  |  |  |  |  |  |  |  |  |
|  | **MFSUL** | 580 | 668 | 672 | 664 | 782 | 528 | 321 | 311 | 312 | 168 | 93 | 76 | --- |  |  |  |  |  |  |  |  |
|  | **MFBEA** | 599 | 687 | 691 | 683 | 801 | 547 | 341 | 330 | 331 | 187 | 112 | 96 | 19 | --- |  |  |  |  |  |  |  |
|  | **MFMAR** | 599 | 687 | 691 | 683 | 801 | 547 | 341 | 330 | 331 | 187 | 112 | 96 | 19 | 0 | --- |  |  |  |  |  |  |
| **Upper Salmon MPG** | **SRLEM** | 577 | 665 | 670 | 661 | 780 | 525 | 319 | 308 | 310 | 165 | 203 | 220 | 296 | 315 | 315 | --- |  |  |  |  |  |
|  | **SRLMA** | 571 | 659 | 663 | 655 | 773 | 519 | 313 | 302 | 303 | 159 | 197 | 214 | 290 | 309 | 309 | 96 | --- |  |  |  |  |
|  | **SREFS** | 669 | 757 | 761 | 753 | 871 | 617 | 411 | 400 | 401 | 257 | 295 | 311 | 388 | 407 | 407 | 194 | 98 | --- |  |  |  |
|  | **SRYFS** | 718 | 806 | 810 | 802 | 921 | 666 | 460 | 449 | 451 | 306 | 344 | 361 | 437 | 456 | 456 | 243 | 147 | 49 | --- |  |  |
|  | **SRVAL** | 732 | 820 | 824 | 816 | 934 | 680 | 473 | 463 | 464 | 320 | 358 | 374 | 451 | 470 | 470 | 257 | 161 | 63 | 27 | --- |  |
|  | **SRUMA** | 740 | 829 | 833 | 824 | 943 | 688 | 482 | 471 | 473 | 328 | 366 | 383 | 459 | 478 | 478 | 265 | 169 | 71 | 36 | 9 | --- |

**Table S5**: Geographic stream network distances (km; adapted from ICTRT 2003) between Major Population Groups from the Snake River system and the Upper Columbia ESU.

| **MPG or ESU** | **Grand Ronde/**  **Imnaha MPG** | **Middle Fork**  **Salmon River MPG** | **South Fork**  **Salmon River MPG** | **Upper**  **Salmon River MPG** | **Upper**  **Columbia ESU** |
| --- | --- | --- | --- | --- | --- |
|  |  |  |  |  |  |
| **Grand Ronde/**  **Imnaha MPG** | --- |  |  |  |  |
| **Middle Fork**  **Salmon River MPG** | 218 | --- |  |  |  |
| **South Fork**  **Salmon River MPG** | 92 | 127 | --- |  |  |
| **Upper Salmon**  **River MPG** | 322 | 104 | 230 | --- |  |
| **Upper Columbia**  **ESU** | 535 | 741 | 614 | 844 | --- |
