# Supplementary material for: Assessing spatial covariance among time series of abundance
Source: Ecol Evol. 2016 Mar 12;6(8):2472–85. doi: 10.1002/ece3.2031 (PMC4789304; doi:10.1002/ece3.2031)
Supplement: Supplementary file 2 — Data S1. R scripts illustrating DFA model fitting. [file ECE3-6-2472-s002.pdf]

Supplement: Fitting DFA models in MARSS

DFA model fitting examples

The MARSS R package (Holmes et al. 2012, 2013) comes with an extensive user guide and introduction to multivariate autoregressive state-space models and modeling. One section of it (Chap. 9) is devoted solely to dynamic factor analysis (DFA) with several examples and code (available with the package, and online: https://cran.r-project.org/web/packages/MARSS/vignettes/UserGuide.pdf).

The following is an illustration of fitting DFA models to Chinook salmon time series of abundance data using the R MARSS package. This example includes models fitted with from 3 to 5 latent variables ($M = 3$, 4, or 5) and with 3 alternative forms of the R-matrix. This matrix is the variance-covariance of the observation model, and this illustration includes a comparison of models with these alternative forms (Holmes et al. 2013): "unconstrained" (variances and covariances are allowed to all differ, all matrix elements are estimated), "diagonal and equal" (one variance value across the diagonal is estimated and no covariance, thus zeros in the off-diagonal elements), and "equal variance covariance" (variance value across the diagonal is shared and estimated, and the population covariances (the off-diagonal elements) are shared and estimated).

The data consist of time series of spawner abundances for the 2010 species status review (available here: https://www.webapps.nwfsc.noaa.gov/apex/f?p=261:HOME, 2010 status review archive). Natural origin spawners were calculated as Spawners x FracWild. We natural log-transformed and then standardized the data (subtracted the mean and divided by the standard deviation). Following instructions in Holmes et al. (2012) to prepare the data for MARSS, we excluded columns and rows other than the actual transformed data (including removing "year" column) and transposed the data so that spawner counts over the years go across the columns. Tucannon River, Pahsimeroi River, and Chamberlain Creek populations, and also the first several years in all time series were omitted because of missing data. Environmental covariates are not included in this illustration but Chap. 12 of the MARSS package user manual provides detailed instructions for adding covariates.

```
# data #
#   data were downloaded from the SPS Salmon Population Summary database,
#   the 2010 status review archive
dat.sps <- read.csv("~/jorgensen/data/R/sps_data.csv", header=FALSE, as.is=TRUE)
sps <- dat.sps[-1,-1]# remove year row at top, names column at left
# log data
log.sps <- log(sps)
# standardize time series
Sigma <- sqrt(apply(log.sps, 1, var, na.rm=TRUE))
y.bar <- apply(log.sps, 1, mean, na.rm=TRUE)
log.sps.z <- ((log.sps-y.bar)*(1/Sigma))
# change input data from data.frame to matrix for MARSS
# because of missings:
#   removing first 8 years, and remove Tucannon, Chamberlain, and Pahsimeroi
dat <- as.matrix(log.sps.z[c(-7, -14, -22), seq(-1, -8, by=-1)])
```

The fitting process produces a considerable amount of screen outputs which we have suppressed (set silent=FALSE to see them). For this illustration, to speed up the model fitting process the number of iterations "maxit" is set to a maximum of 500, and as a consequence many parameter values have not yet converged. This code took <10 min. on a MacOS desktop. When doing model fitting to reach convergence maxit should be set higher. See Chap. 9 on DFA model fitting and other sections of the package user guide for more details on options, procedures, and guidance for setting the number of iterations.

```r
library(MARSS)

# model #
# specify model structures to compare:
#    fit models with M=3, 4, and 5 latent variables across
#    3 different R-matrix structures
levels.R<-c("unconstrained", "diagonal and equal", "equalvarcov")
N.ts <- dim(dat)[1]
A <- matrix(0, nrow=N.ts, ncol=1)
# set the min & max number of latent variables estimated
M.min <- 3
M.max <- 5

# set counter for model results
 cnt <- 1

# total num of models to test
n.set <- length(levels.R)*(M.max - M.min + 1)

# create empty list to store model results
mod.fit <- vector("list", n.set)

#  Note: This will take a while to run.....

  for(R in levels.R){# looping over R structures
    #    store name of R for later
    r.name <- R
    for(m in M.min:M.max){# looping over number of latent variables, or trends
        Z <- matrix(list(), N.ts, m)
        for(i in seq(N.ts)) {Z[i,] <- paste(i, seq(m), sep="")}
        if(m > 1) {for(i in 1:(m-1)){Z[i, (i+1):m] <- 0}}
        x0 <- U <- matrix(0, m, 1)
        Q <- B <- diag(1, m)
        V0 <- diag(5, m)
        dfa.model <- list(A="zero", R=R, m=m)
        # fit DFA & store results
        mod.fit[[cnt]] <- MARSS(dat,
                                model=dfa.model, z.score=TRUE, form="dfa",
                                control=list(maxit=500, conv.test.slope.tol=0.05,
                                             demean.states=TRUE,
                                             safe=TRUE,
                                             trace=0),silent=TRUE)
        mod.fit[[cnt]]$R <- r.name
        mod.fit[[cnt]]$M <- m
        cnt <- cnt + 1

    } #ending m loop
  } # ending R loop
```

The following are the results from the above code.

```r
# table of model results
mod.sel.tbl <- data.frame(
```

```r
  num=seq(1:length(mod.fit)),
  K=sapply(mod.fit,
           function(x) if (is.null(x[["num.params"]])) 0 else x[["num.params"]]),
  R=sapply(mod.fit, function(x) x[["R"]]),
  M=sapply(mod.fit, function(x) x[["M"]])
)
  AICc.results <- sapply(mod.fit,
                         function(x) if (is.null(x[["AICc"]])) 9999 else x[["AICc"]])

# calculate delta.AICc
mod.sel.tbl$delta.AICc[AICc.results < 9999] <- (AICc.results[AICc.results < 9999]
                                                 - min(AICc.results[AICc.results < 9999]))

# print table
print(mod.sel.tbl)
```

```
##   num   K                  R M delta.AICc
## 1   1   0      unconstrained 3         NA
## 2   2   0      unconstrained 4         NA
## 3   3   0      unconstrained 5         NA
## 4   4  70 diagonal and equal 3 28.2877208
## 5   5  91 diagonal and equal 4 13.9505348
## 6   6 111 diagonal and equal 5 10.4779041
## 7   7  71        equalvarcov 3  4.1651905
## 8   8  92        equalvarcov 4  0.2624647
## 9   9 112        equalvarcov 5  0.0000000
```

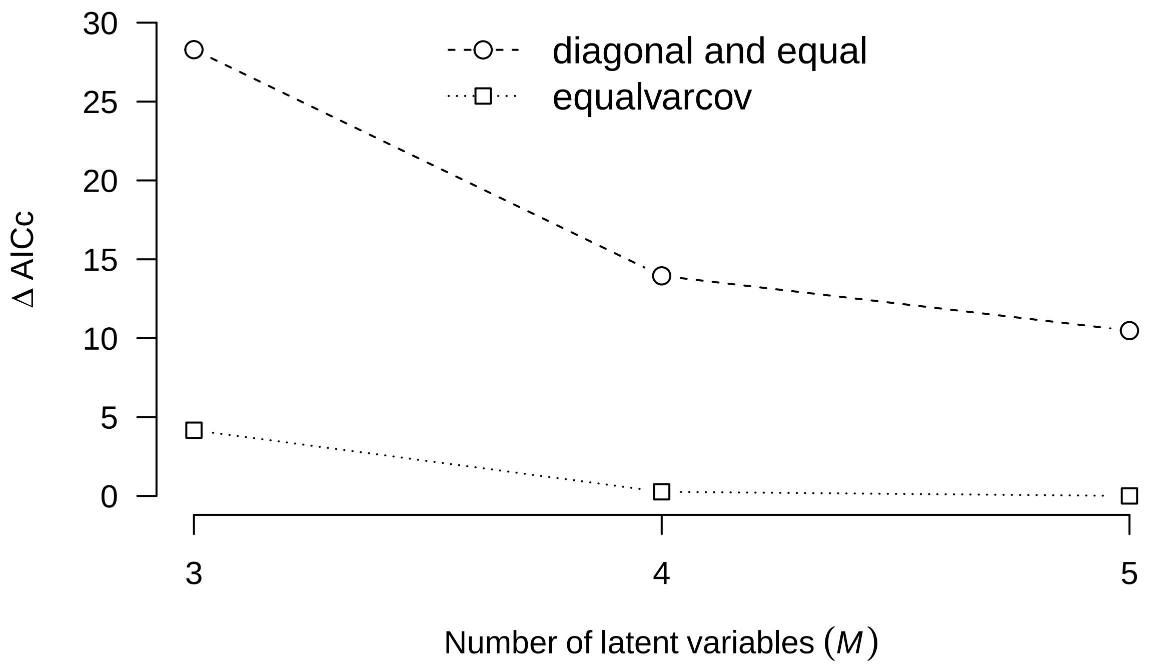

The table includes model index ("num"), the number of parameters estimated ("K"), the form of the R matrix ("R"), the number of latent variables, M ("M"), followed by a measure of data support for each model (ΔAICc). The results from the table are also depicted in the plot. There was more data support for the R-matrix taking the "equal variance covariance" form, where both the variance and covariance is shared

across the populations and each are estimated in the model fitting. MARSS was not able to reach stable solutions with R set to "unconstrained" (ΔAICc = NA denotes models that were not fit).

We can plot the fits for the best model (in this illustration) along with the data. These functions return the fitted values and $\pm$ (1-α)% confidence intervals.

```
MARSShatyt = function( MLEobj ) {
  #
  modelObj = MLEobj[["marss"]]
  if(!is.null(MLEobj[["kf"]])){ kfList = MLEobj$kf
  }else{ kfList=MARSSkf(MLEobj) }
  model.dims=attr(modelObj,"model.dims")
  n=model.dims$data[1]; TT=model.dims$data[2]; m=model.dims$x[1]

  #create the YM matrix
  YM=matrix(as.numeric(!is.na(modelObj[["data"]])),n,TT)
  #Make sure the missing vals in y are zeroed out if there are any
  y=modelObj$data
  y[YM==0]=0

  #set-up matrices for hatxt for 1:TT and 1:TT-1
  IIz=list()
  IIz$V0=MARSS:::makediag(as.numeric(MARSS:::takediag(MARSS:::parmat(MLEobj,
                                                                "V0", t=1)$V0)==0),m)
  #
  hatxt=kfList$xtT       #1:TT
  hatxtt1=kfList$xtt1      #1:TT
  E.x0 = (diag(1,m)-IIz$V0)%*%kfList$x0T+IIz$V0%*%MARSS:::parmat(MLEobj,
                                                            "x0", t=1)$x0  #0:T-1
  hatxt1=cbind(E.x0,kfList$xtT[,1:(TT-1),drop=FALSE])
  hatxtp=cbind(kfList$xtT[,2:TT,drop=FALSE],NA)
  hatVt=kfList$VtT
  hatVtt1=kfList$Vtt1T
  hatVtpt = array(NA,dim=dim(kfList$Vtt1T))
  hatVtpt[,,1:(TT-1)] = kfList$Vtt1T[,,2:TT,drop=FALSE]

  msg=NULL

  #Construct needed identity matrices
  I.n = diag(1,n)

  #Note diff in param names from S&S;B=Phi, Z=A, A not in S&S
  time.varying = c(); pari=list()
  for(elem in c("R","Z","A")){  #only params needed for this function
    if( model.dims[[elem]][3] == 1 ){  #not time-varying
      pari[[elem]]=MARSS:::parmat(MLEobj, elem, t=1)[[elem]]
      if(elem=="R"){
        if(length(pari$R)==1) diag.R=unname(pari$R) else {
          diag.R = MARSS:::takediag(unname(pari$R))}
         is.R.diagonal = all(pari$R[!diag(nrow(pari$R))] == 0) # = isDiagonal(pari$R)
      }
    }else{ time.varying = c(time.varying, elem) } #which elements are time varying
  }#end for loop over elem
```

```r
#initialize - these are for the forward, Kalman, filter
# for notation purposes, 't' represents current point in time,
# 'TT' represents the length of the series
#
hatyt = hatytt1 = matrix(0,n,TT)
hatOt = array(0,dim=c(n,n,TT))
hatyxt = hatyxtt1 = hatyxttp = array(0,dim=c(n,m,TT))

for (t in 1:TT) {
  for(elem in time.varying){
    pari[[elem]]=MARSS:::parmat(MLEobj, elem, t=t)[[elem]]
    if(elem=="R"){
      if(length(pari$R)==1) diag.R=unname(pari$R) else {
        diag.R = MARSS:::takediag(unname(pari$R))}
      is.R.diagonal = all(pari$R[!diag(nrow(pari$R))] == 0)
    }
  }
  if(all(YM[,t]==1)){  #none missing
    hatyt[,t]=y[,t,drop=FALSE]
    hatytt1[,t]=pari$Z%*%hatxtt1[,t,drop=FALSE]+pari$A
    hatOt[,,t]=tcrossprod(hatyt[,t,drop=FALSE]) #
    hatyxt[,,t]=tcrossprod(hatyt[,t,drop=FALSE], hatxt[,t,drop=FALSE])
    hatyxtt1[,,t]=tcrossprod(hatyt[,t,drop=FALSE], hatxt1[,t,drop=FALSE])
    hatyxttp[,,t]=tcrossprod(hatyt[,t,drop=FALSE], hatxtp[,t,drop=FALSE])
  }else{
    I.2 = I.r = I.n;
    I.2[YM[,t]==1,]=0 #1 if YM=0 and 0 if YM=1
    I.r[YM[,t]==0 | diag.R==0,]=0   #if Y missing or R = 0, then 0
    Delta.r=I.n
    if(is.R.diagonal) Delta.r = I.n-I.r
    if(!is.R.diagonal && any(YM[,t]==1 & diag.R!=0)){
      mho.r = I.r[YM[,t]==1 & diag.R!=0,,drop=FALSE]
      t.mho.r = I.r[,YM[,t]==1 & diag.R!=0,drop=FALSE]
      Rinv = try(chol(mho.r%*%pari$R%*%t.mho.r))
      #Catch errors before entering chol2inv
      if(class(Rinv)=="try-error") {
        return(list(ok=FALSE, errors="Stopped in MARSShatyt: chol(R) error.\n" ))
      }
      Rinv=chol2inv(Rinv)
      Delta.r = I.n- pari$R%*%t.mho.r%*%Rinv%*%mho.r
    }
    hatyt[,t] = (y[,t,drop=FALSE]
                 - Delta.r%*%(y[,t,drop=FALSE]-pari$Z%*%hatxt[,t,drop=FALSE]-pari$A))
    hatytt1[,t]=pari$Z%*%hatxtt1[,t,drop=FALSE]+pari$A
    t.DZ = matrix(Delta.r%*%pari$Z,m,n,byrow=TRUE)
    hatOt[,,t] = (I.2%*%(Delta.r%*%pari$R+Delta.r%*%pari$Z%*%hatVt[,,t]%*%t.DZ)%*%I.2
                  + tcrossprod(hatyt[,t,drop=FALSE]))
    hatyxt[,,t] = (tcrossprod(hatyt[,t,drop=FALSE],
                              hatxt[,t,drop=FALSE])
                   + Delta.r%*%pari$Z%*%hatVt[,,t])
    hatyxtt1[,,t] = (tcrossprod(hatyt[,t,drop=FALSE],
                                hatxt1[,t,drop=FALSE])
                     + Delta.r%*%pari$Z%*%hatVtt1[,,t])
```

```
      hatyxttp[,,t] = (tcrossprod(hatyt[,t,drop=FALSE],
                                   hatxtp[,t,drop=FALSE])
                        + Delta.r%*%pari$Z%*%t(hatVtpt[,,t]))
    }
  } #for loop over time
  rtn.list=list(ytT = hatyt, OtT = hatOt, yxtT=hatyxt, yxt1T=hatyxtt1,
                yxttpT = hatyxttp, ytt1 = hatytt1)
  return(c(rtn.list,list(ok=TRUE, errors = msg)))
}

fittedDFA <- function(MLEobj,alpha=0.05) {
  # empty list for results
    fits <- list()
    # model params
  par.mat <- coef(MLEobj, type="matrix")
    # extra stuff for var() calcs
    Ey <- MARSShatyt(MLEobj)
    # model params
    ZZ <- coef(MLEobj, type="matrix")$Z
    # number of obs ts
    nn <- dim(Ey$ytT)[1]
    # number of time steps
    TT <- dim(Ey$ytT)[2]
    # get the inverse of the rotation matrix
    H.inv <- varimax(ZZ)$rotmat
    # model expectation
    fits$ex <- ZZ %*% H.inv %*% MLEobj$states + matrix(par.mat$A,nn,TT)
    # Var in model fits
    VtT <- MARSSkfss(MLEobj)$VtT
    VV <- NULL
    for(tt in 1:TT) {
        RZVZ <- par.mat$R - ZZ%*%VtT[,,tt]%*%t(ZZ)
        SS <- (Ey$yxtT[,,tt]
               - Ey$ytT[,tt,drop=FALSE] %*% t(MLEobj$states[,tt,drop=FALSE]))
        VV <- cbind(VV,diag(RZVZ + SS%*%t(ZZ) + ZZ%*%t(SS)))
    }
    SE <- sqrt(VV)
    # upper (1-alpha)% CI
    fits$up <- qnorm(1-alpha/2)*SE + fits$ex
    # lower (1-alpha)% CI
    fits$lo <- qnorm(alpha/2)*SE + fits$ex
    return(fits)
}
```

```
my.fits <- fittedDFA(mod.fit[[9]])
# population names
spp2 <- dat.sps[c(-1, -8, -15, -23),1]
spp2[c(16,17)] <- c("Salmon River Lower", "Salmon River Upper")

par(mfrow=c(5,5), mar=c(3,4,1.5,0.5), oma=c(0.4,1,1,1),las=1,tcl=0.5)
for(i in 1:length(spp2)){
  plot(dat[i,], xlab="", ylab="", bty="L", xaxt="n", ylim=c(-4,4),
       pch=16, col=NA)
```

```
  axis(1, at=seq(0, 53, by=10), labels=seq(1957, 2009, by=10))
  polygon(x=c(1:53, 53:1), y=c(my.fits$up[i,],rev(my.fits$lo[i,])),
          border=NA,col="darkgray")
  points(dat[i,], col="black")
  lines(my.fits$ex[i,], lwd=2)
  title(substr(spp2[i], 1, 16))
}
mtext(side=2, outer=TRUE, line=-1,"Standardized log abundance", las=3)
```

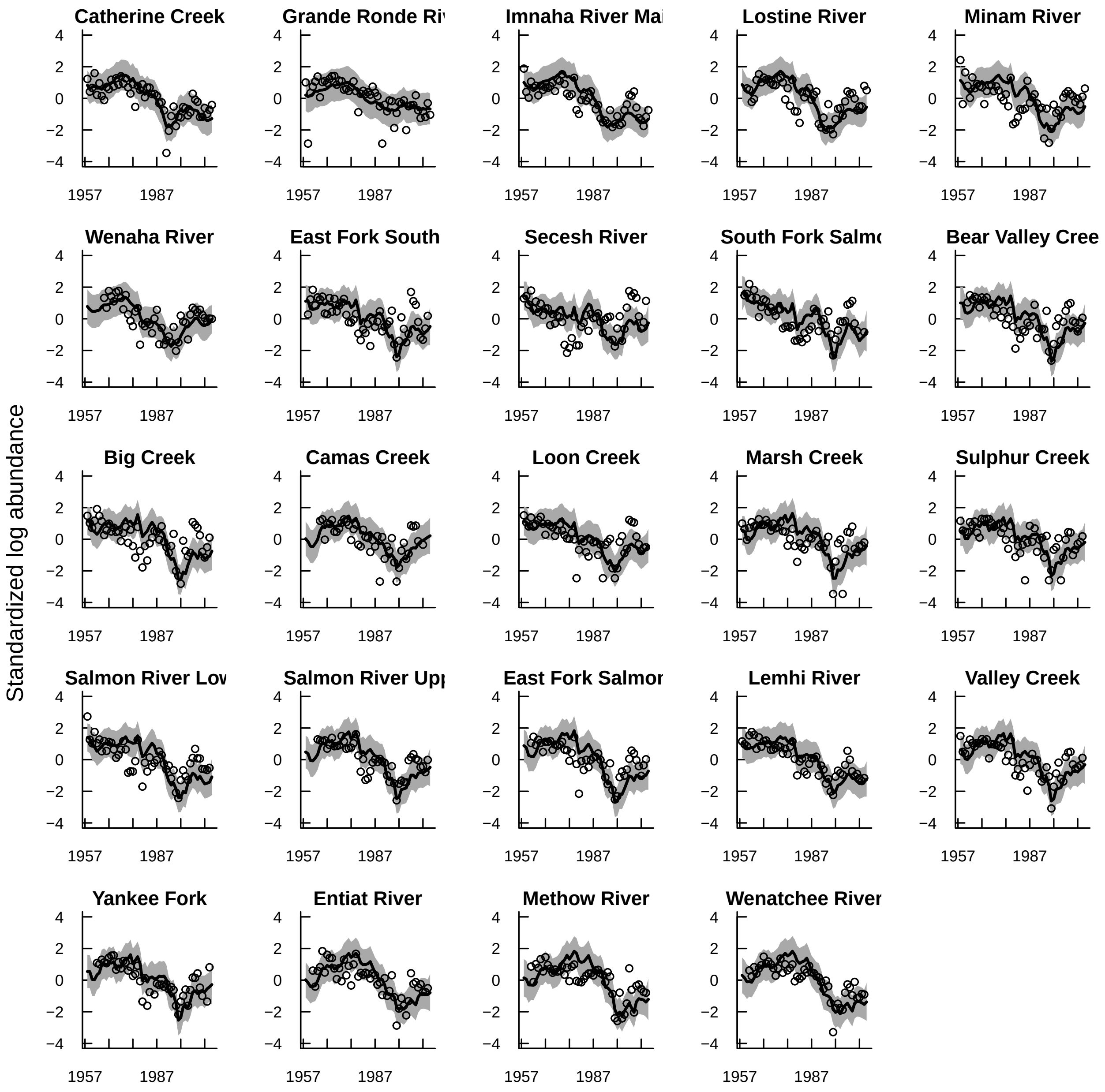

References

Holmes, E.E., Ward, E.J. and Wills, K. 2012. MARSS: Multivariate autoregressive state-space models for analyzing time-series data. R Journal, 4, 11-19.

Holmes, E.E., Ward, E.J. and Wills, K. 2013. MARSS: Multivariate Autoregressive State-Space Modeling. R package version 3.9.
